# Case report: Thrombolysis in patients with acute ischemic stroke and cerebral cavernous malformation

**DOI:** 10.3389/fneur.2023.1281412

**Published:** 2023-12-18

**Authors:** Jie Lin, Xiongpeng Weng, Jing Zheng, Saizhen Wu, Qiongqiong Bao, Feifei Peng, Yanbin Huang

**Affiliations:** ^1^Department of Neurology, Yueqing People’s Hospital, Wenzhou, China; ^2^Department of Neurology, Affiliated Huangyan Hospital of Wenzhou Medical University, Taizhou First People's Hospital, Taizhou, China

**Keywords:** thrombolysis, intravenous tissue plasminogen activator, acute stroke, cerebral cavernous malformation, case series, literature review

## Abstract

**Background:**

Cerebral cavernous malformation (CCM) is a rare disease associated with a latent risk of intracranial hemorrhage. However, due to limited evidence, the safety of recommending intravenous tissue plasminogen activators for patients with acute stroke and CCM remains uncertain.

**Methods:**

Our study identified five patients with acute stroke and CCM treated between 2017 and 2023 across two hospitals. A comprehensive literature review was conducted, incorporating three similar case reports and two retrospective studies.

**Results:**

Among 30 patients reviewed, three exhibited symptomatic intracranial hemorrhage, two of whom were women. Additionally, three patients presented with calcification in their CCM, with two experiencing symptomatic intracranial hemorrhage.

**Conclusion:**

The observed incidence of symptomatic intracranial hemorrhage following intravenous tissue plasminogen activator administration appears to be elevated in patients with CCM. Therefore, before thrombolysis, a thorough evaluation of personalized risk–benefit ratios is crucial. Furthermore, conducting further research involving multiple centers and larger sample sizes is imperative to advance our understanding in this area, especially in identifying hemorrhage risk factors.

## Introduction

Acute ischemic stroke (AIS) is a prominent cause of death and disability globally. The approach to AIS treatment underwent a transformation worldwide following the introduction of intravenous tissue plasminogen activator (IV-tPA) in clinical settings by the National Institute of Neurological Disorders and Stroke study in 1995 (1). IV-tPA remains the sole beneficial drug for patients with AIS within 4.5 h of symptom onset. During the past decades, substantial real-world experience has accumulated regarding IV-tPA’s efficacy and safety across varied clinical scenarios, rendering some initial contraindications suitable for IV-tPA (2). Nonetheless, because of the risk of hemorrhagic complications, particularly symptomatic intracranial hemorrhage (sICH), the use of IV-tPA remains uncertain in certain cases. According to a recent guideline by the American Heart Association/American Stroke Association (3), the utility and risks of IV-tPA treatment for patients with AIS who have known unruptured and untreated intracranial vascular malformations still lack definitive establishment due to limited data (COR IIb; LOE C-LD).

Cerebral cavernous malformation (CCM) represents an intracranial vascular malformation with a low incidence, reported at 0.5%. Moreover, 95% of cases were asymptomatic, as revealed in a retrospective analysis involving 24,535 autopsies (4). Because of its rarity, only a handful of reports address the use of IV-tPA in patients with AIS and CCM. To evaluate the safety of IV-tPA in patients with AIS and CCM, we presented five cases treated with IV-tPA, all of whom demonstrated favorable outcomes. Additionally, our literature review encompassed three similar case reports and two retrospective studies (5–9).

## Methods

From January 1, 2017, to April 1, 2023, we conducted a comprehensive review of all patients with AIS who underwent IV-tPA treatment across our two hospitals. Within this cohort, five patients with CCMs were identified.

**Figure 1:**
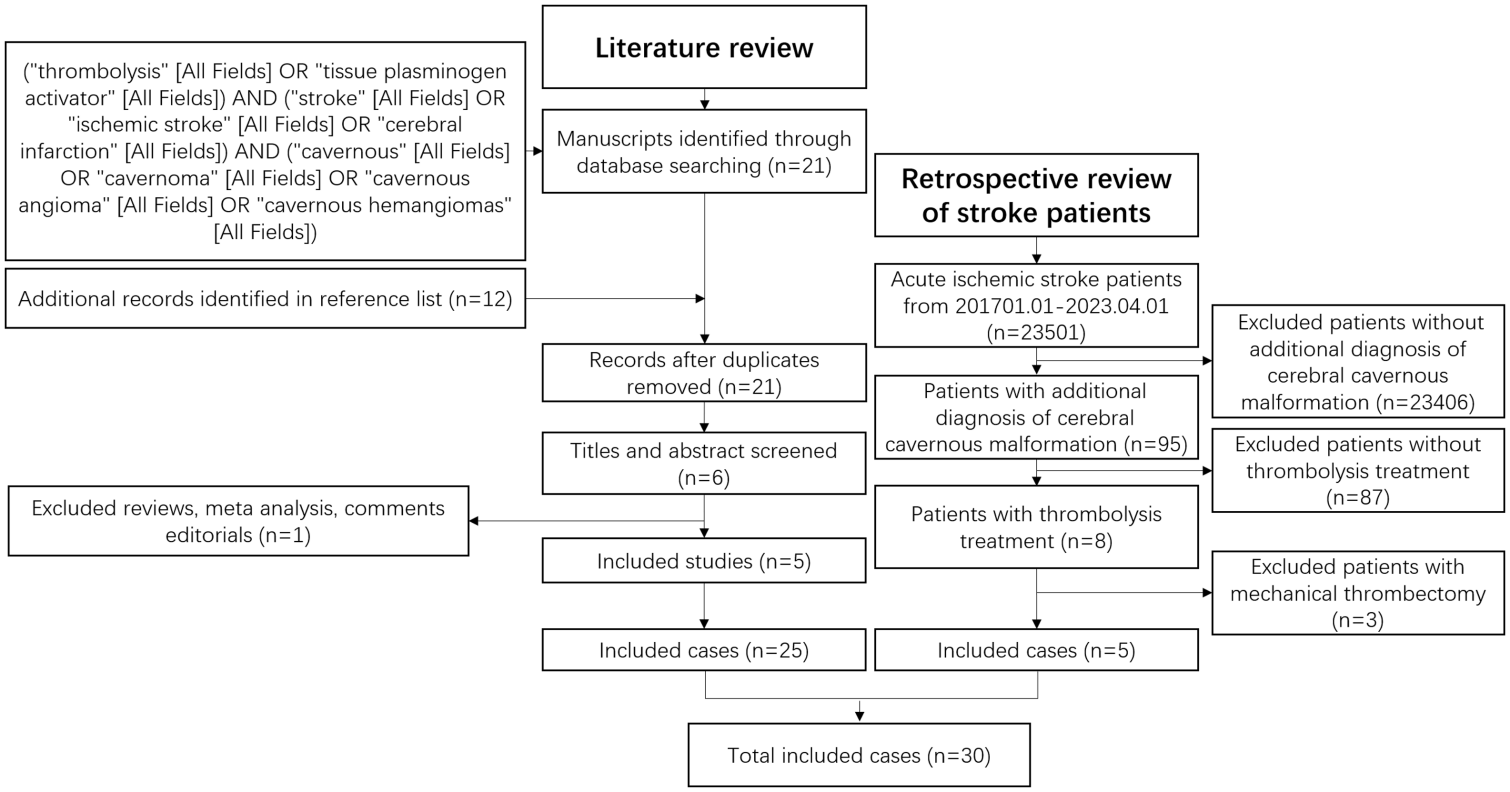
Literature review and retrospective review strategy.

A systematic search was conducted using PubMed with specific keywords: (“thrombolysis” [All Fields] OR “tissue plasminogen activator” [All Fields]) AND (“stroke” [All Fields] OR “ischemic stroke” [All Fields] OR “cerebral infarction” [All Fields]) AND (“cavernous” [All Fields] OR “cavernoma” [All Fields] OR “cavernous angioma” [All Fields] OR “cavernous hemangiomas” [All Fields]). Relevant studies were selected based on title and abstract evaluation, and references were also examined. Methodology. Figure 1 outlines the methodology employed for this systematic review.

## Results

### Case series

#### Case report 1

A 52-year-old male patient with a history of hypertension was admitted to our neurology department due to numbness and slight motor weakness in his right hand and leg. His blood pressure on arrival was 207/119 mmHg. Neurological examination revealed right-sided hemiparesis and hemianesthesia, resulting in a National Institutes of Health Neurological Deficit Score (NIHSS) score of 2. A computed tomography (CT) scan (Supplementary Figure 1A) showed no intracranial hemorrhage (ICH) at presentation. Within the treatment window and with no contraindications except hypertension, IV-tPA (0.9 mg/kg) was initiated 108 min after symptom onset, following a blood pressure reduction to 177/99 mmHg. A follow-up CT scan after 24 h revealed no evidence of ICH. Subsequently, antiplatelet therapy was commenced. A magnetic resonance imaging (MRI) conducted 72 h later detected a CCM in the left frontal lobe (Supplementary Figure 1B) along with scattered AIS lesions in various brain regions (Supplementary Figure 1C). His neurological deficits completely resolved during hospitalization (NIHSS 0), and he was discharged after 10 days, continuing antiplatelet treatment. No complaints were reported during the follow-up.

#### Case report 2

A 58-year-old male patient with a 10-year history of hypertension and 20 years of smoking presented with a sudden onset of left motor weakness and unclear speech. On admission, the patient’s vital signs were stable. Physical examination revealed left hemiplegia, facial paralysis, and dysarthria with a NIHSS score of 7. A CT scan (Supplementary Figure 2A) was performed, confirming eligibility for IV-tPA (0.9 mg/kg) 137 min after symptom onset. Progressive improvement was observed during treatment, and he fully recovered the following day. An MRI after 24 h revealed ischemic lesions in the right basal ganglia and a 4 × 6 mm lesion in the left temporal lobe, confirming the presence of a CCM (Supplementary Figures 2B,C). He was initiated on antiplatelet therapy (aspirin 0.2 g/qd) before discharge. He was discharged on the 13th day, continuing aspirin (0.1 g/qd regularly) without any reported intracerebral hemorrhage during follow-up.

#### Case report 3

A 69-year-old male patient, with a 4-year history of atrial fibrillation without anticoagulation therapy, was admitted to our hospital due to difficulty walking and speaking. On arrival, his vital signs were stable except for his heart rate. A neurological examination revealed mild drowsiness, aphasia, dysarthria, horizontal gaze palsy, left facial paresis, left hemiplegia, and hemidysesthesia. His NIHSS score was 8. A CT scan immediately revealed a calcification lesion in the right parietal lobe (Supplementary Figure 3A), with no evidence of ICH. After excluding contraindications, IV-tPA was administered at a dosage of 0.9 mg/kg, 250 min after symptom onset. His symptoms began to improve shortly after treatment. The following day, an MRI indicated scattered ischemic lesions in the right frontal lobe, temporal lobe, and basal ganglia (Supplementary Figure 3B). A CCM was also identified in the right parietal lobe (Supplementary Figure 3C). Following a 2-week hospitalization, a subsequent CT scan showed no signs of ICH. Subsequently, anticoagulation therapy was initiated. At discharge, his NIHSS score had improved to 2, with only mild aphasia and left hemiparesis. Throughout the follow-up period, there were no reported complaints while continuing anticoagulation therapy.

#### Case report 4

A 63-year-old male patient, with a 10-year history of managing hypertension and type 2 diabetes, was admitted due to unclear speech and motor weakness in the right leg and hand. During the physical examination, mild right facial paralysis, aphasia, and right hemiparesis were observed, resulting in an NIHSS score of 5. His vital signs remained stable. A CT scan revealed a suspicious lesion in the left basal ganglia (Supplementary Figure 4A). After excluding contraindications, IV-tPA was administered 57 min after symptom onset. His symptoms began to improve, and the following day, another CT scan showed no signs of ICH. Aspirin was then initiated as antiplatelet therapy. During his hospital stay, an MRI and susceptibility-weighted imaging detected lesions from an ischemic stroke in the left pons and a CCM in the left basal ganglia (Supplementary Figures 4B–D). After 10 days in the hospital, he was discharged with mild residual right facial paresis, aphasia, and right hemiparesis, with an NIHSS score of 3. Aspirin therapy for antiplatelet purposes was continued during the follow-up where no ICH was reported.

#### Case report 5

An 80-year-old male patient, with a 6-year history of hypertension and diabetes, arrived at our hospital with left-sided motor weakness and difficulty speaking clearly. Upon arrival, his vital signs were stable. Physical examination revealed mild left facial paralysis, dysarthria, and left-sided hemiparesis, resulting in an NIHSS score of 4. A CT scan (Supplementary Figure 5A) was performed, and 129 min after symptom onset, IV-tPA was administered following the exclusion of contraindications. The subsequent day, an MRI revealed a 7 mm × 8 mm CCM in the right periventricular area (Supplementary Figure 5B). Scattered ischemic stroke lesions were also found in the right temporal and occipital lobes (Supplementary Figure 5C). Throughout the 9-day hospitalization, antiplatelet therapy with aspirin 0.1 g/qd was initiated. His symptoms exhibited slight improvement, with an NIHSS score of 2. During the follow-up period, no complaints were reported.

## Literature review

The demographic and clinical features of 17 patients were extracted and are presented in Table 1. Among these, three cases had documented sICH, one of which involved hemorrhage occurring in an ischemic lesion. Additionally, three cases demonstrated calcification in CCM, with two of these presenting sICH. Of the total patients, only seven were women, two of whom experienced sICH. In the retrospective study involving 13 patients (Table 2), there was no documented sICH. Unfortunately, neither the number of patients with ICH nor the demographic and clinical features were reported.

**Table 1:** Demographic and clinical manifestations.

Number (Age/ Sex)	Author	Year	CCM Type	Number of CCM	Known CCM	CCM site	Initial NIHSS	History	OTT	Dose	Calcification in CCM	sICH	ICH	ICH in CMM	Prognosis: mRS
1 (79/♂)	Henninger	2007	I	1	Yes	Left temporal lobe	11	HP, dyslipidemia, sleep apnea, coronary artery bypass graft, dissection of the right coronary artery after cardiac catheterization, AF, smoking	138	–	No	No	No	No	0
2 (58/♂)	Gattringer	2013	II	1	No	Fronto-parietal white matter	6	No	150	–	Yes	Yes	Yes	Yes	4
3 (76/♀)	Guillon	2014	–	1	No	Pontine	14	Type 2 diabetes, HP, myocardial infarction, and a pacemaker was implanted.	150	0.9 mg/kg	Yes	Yes	Yes	Yes	6
4 (52/♂)	Case 1	–	I, III	2	No	Left frontal lobe	2	HP	108	0.9 mg/kg	No	No	No	No	0
5 (58/♂)	Case 2	–	III	1	No	Left frontal lobe	7	HP, smoking	137	0.9 mg/kg	No	No	No	No	0
6 (69/♂)	Case 3	–	II	3	No	Right parietal lobe	8	AF	250	0.9 mg/kg	Yes	No	No	No	1
7 (63/♂)	Case 4	–	III	1	No	Left basal ganglia	5	HP, type 2 diabetes	57	0.9 mg/kg	No	No	No	No	1
8 (80/♂)	Case 5	–	I	1	No	Right periventricular	4	HP, type 2 diabetes	109	0.9 mg/kg	No	No	No	No	1
9 (93/♀)	Hebun	2014	III	2	–	Left temporal, left cerebellar	10	–	–	–	–	Yes	Yes	No	6
10 (81/♂)	Hebun	2014	I, III	3	–	Left occipital, two left periventricular	2	–	–	–	–	No	Yes	Yes	1
11 (87/♀)	Hebun	2014	III	1	–	Pons	7	–	–	–	–	No	No	No	0
12 (89/♀)	Hebun	2014	III	1	–	Right temporobasal	15	–	–	–	–	No	No	No	5
13 (73/♀)	Hebun	2014	I	1	–	Right temporal	7	–	–	–	–	No	No	No	1
14 (90/♀)	Hebun	2014	III	1	–	Pons	18	–	–	–	–	No	No	No	4
15 (60/♀)	Hebun	2014	I	1	–	Left parietal	5	–	–	–	–	No	No	No	3
16 (79/♂)	Hebun	2014	III	1	–	Right frontal	8	–	–	–	–	No	No	No	0
17 (72/♂)	Hebun	2014	III	1	–	Pons	4	–	–	–	–	No	No	No	4

AF, atrial fibrillation; CMM, cerebral cavernous malformation; HP, Hypertension; ICH, intracranial hemorrhage; mRS, Modified Rankin Scale; NIHSS, National Institutes of Health Neurological Deficit Score; OTT, onset-to-treatment time; sICH, symptomatic intracranial hemorrhage; −, without relative information.

**Table 2:** sICH in two retrospective studies.

Number	Author	Year	Number of patients	ICH	sICH
1	Christopher	2018	13	–	0
2	Hebun	2014	9	2	1

ICH, intracranial hemorrhage; sICH, symptomatic intracranial hemorrhage; −, without relative information.

In total, 30 patients were included, among whom three (10.00%) experienced sICH. As the specific number of patients with ICH was not specified in the retrospective study involving 13 patients, the ratio of patients with ICH was calculated as (4/17, 23.53%). A simple comparison between patients with sICH and those with non-sICH was conducted among the 17 patients, as depicted in Table 3. Patients with sICH tended to be older, with a higher percentage being women and had calcified lesions in CCM, a higher initial NIHSS, and a longer onset-to-treatment time. Notably, the number and type of CCM appeared similar between patients with sICH and those with non-sICH.

**Table 3:** Comparison between sICH and non-sICH.

	sICH	Non-sICH
Age (years old)	75.67 ± 14.30	73.71 ± 11.56
Sex (female)	2/3 (66.67%)	5/14 (35.71%)
Initial NIHSS	10 ± 3.27	7.36 ± 4.45
OTT (min)	150 ± 0	83 ± 58.75
Number of CCM	1.33 ± 0.47	1.36 ± 0.72
CCM Type I	1/2 (50%)	6/14 (42.86%)
CCM Type II	0/2 (0%)	1/14 (7.14%)
CCM Type III	1/2 (50%)	9/14 (64.23%)
Calcification in CCM	2/2 (100%)	1/7 (14.29%)
ICH in CMM	2/3 (66.67%)	1/14 (7.14%)
Prognosis: mRS	5.33	1.5

CCM, cerebral cavernous malformation; ICH, intracranial hemorrhage; mRS, Modified Rankin Scale; OTT, onset-to-treatment time; sICH, symptomatic intracranial hemorrhage.

The analyses were conducted on patients who had available data.

## Discussion

To the best of our knowledge, this represents the first case series involving the administration of IV-tPA in patients with AIS and CCM. CCM is a neurosystemic vascular malformation predominantly found in the brain, spinal cord, or even the dura. It typically remains asymptomatic, as observed in our patients, while a few patients may receive a diagnosis due to symptoms such as seizures, focal neurologic deficits, or acute headaches, which might be associated with ICH (10). CCM is an important cause of ICH, with an overall reported hemorrhage rate of 2.19% per year in an 8-year retrospective study involving 133 patients (11). The risk of hemorrhage associated with CCM appears higher in familial cases than in sporadic CCM, as well as in women (12), those located in the brainstem (13), and post-hemorrhage cases (12). Interestingly, neither the size nor the number of lesions poses a potential risk for hemorrhage (12).

In clinical practice, the risk of hemorrhage in patients with CCM treated with antithrombotic agents (anticoagulants or antiplatelet agents) seems to be low. Additionally, our patients received anticoagulants or antiplatelet treatments post-discharge without reporting any hemorrhage during the follow-up period. Cohort studies have shown that administering antithrombotic agents did not escalate the incidence of CCM-associated ICH (14). Furthermore, some researchers have suggested that antithrombotic therapy could potentially reduce the occurrence of ICH in patients with CCM (15). This implies the reasonable use of routine antithrombotic therapy in patients with CCM.

To date, studies in animal models of CCM have demonstrated the loss of cell–cell junctions and inter-brain endothelial junctions (16). Notably, alterations in the expressions of VE-cadherin, claudin-5, and tight junction protein 1 have been observed in hemorrhagic CCM. Elevated levels of metalloproteinases and oxygen-free radicals have also been confirmed in CCM, potentially causing damage to vascular matrix stability (17, 18). Moreover, increased cytoskeleton contractility has been identified in low-flow areas of CCM endothelium (19–25). The administration of IV-tPA induces local thrombolysis by converting plasminogen into plasmin, which subsequently degrades fibrin into fibrin split products. However, IV-tPA-associated ICH is linked to coagulopathy and disruption of the blood–brain barrier. Therefore, theoretically, IV-tPA might globally affect coagulation in the entire circulatory system, including CCM vessels, potentially leading to ICH in CCM. Only three cases and two retrospective studies on IV-tPA administration in patients with stroke and CCM have been reported (5–9), among which three patients (10.00%) experienced sICH. Two sICH cases occurred in CCM, while one was found in an ischemic lesion. A study involving 2,451 patients reported a 3.5% sICH occurrence rate after thrombolytic therapy (26). Notably, the sICH ratio appears relatively higher in patients with CCM than in patients with regular stroke (10.00% vs. 3.50%), consistent with the results of another study (11.11% vs. 3.23%) (8).

Among the three patients with sICH, two had calcifications in CCM, while only three patients in total exhibited calcification. This suggests that patients with calcific CCM might have a higher susceptibility to sICH after IV-tPA. Researchers have associated calcification in CCM with recurrent thrombosis (27). Furthermore, alterations in the composition and degeneration of the basement membrane have been described in calcific vasculature, with astrocytes around calcifications showing positivity for 2-ω-carboxyethylpyrrole, indicative of inflammation and oxidative stress (28). Such alterations in calcification exacerbate inter-brain endothelial junctions. Additionally, other studies have highlighted being a woman as a risk factor for hemorrhage (12). Estrogen receptors present in CCM make it hormonally responsive (29, 30). Pregnant patients have exhibited more aggressive CCM manifestations (30). In this context, two of the three sICH cases were women (66.67% vs. 35.71%). However, due to the small sample size in our study, we were unable to conduct further analyses such as chi-square tests or linear regression to definitively determine sICH risk factors.

In clinical practice, it is crucial for practitioners to evaluate the risk–benefit ratio of IV-tPA for each patient individually. Patients with seemingly higher hemorrhagic risks, such as those with calcification, female sex, or with limited benefit from IV-tPA, may consider mechanical revascularization. Although we discussed potential hemorrhage risk factors for patients with CCM, these remain theoretical. Hence, identifying hemorrhage risk factors remains important. If feasible, performing MRI in patients with suspected CCM before thrombolysis, especially in those with identified hemorrhage risk factors, might be prudent.

The primary limitation of our study is the small sample size. This limitation may introduce statistical errors, cautioning against broad generalizations of our findings. Furthermore, due to the limited sample, we were unable to perform additional analyses, such as linear regression, on our dataset. Additionally, several intriguing questions regarding the prediction of hemorrhage risk remain unanswered. Moreover, the retrospective nature of our research design represents an inherent limitation. Lastly, incomplete patient information within the systematic review and potential publication/reporting bias might have influenced our findings.

## Conclusion

In summary, the probability of hemorrhage after IV-tPA appears to be high in patients with CCM. Assessing personalized risk–benefit ratios based on a multidisciplinary approach is crucial in clinical decision-making. Considering mechanical revascularization, especially for patients with limited risk–benefit, might be prudent. However, further research involving multiple centers and a larger sample is essential. Identifying hemorrhage risk factors in patients with CCM remains an important objective.

## Data availability statement

The original contributions presented in the study are included in the article/Supplementary material, further inquiries can be directed to the corresponding author.

## Ethics statement

Written informed consent was obtained from the individual(s) for the publication of any potentially identifiable images or data included in this article.

## Author contributions

JL: Methodology, Project administration, Writing – original draft. XW: Methodology, Writing – original draft. JZ: Methodology, Writing – original draft. SW: Methodology, Writing – original draft. QB: Methodology, Writing – original draft. FP: Methodology, Writing – original draft. YH: Project administration, Supervision, Writing – review & editing.
