# Supplementary material for: Case report: Thrombolysis in patients with acute ischemic stroke and cerebral cavernous malformation
Source: Front Neurol. 2023 Dec 18;14:1281412. doi: 10.3389/fneur.2023.1281412 (PMC10758226; doi:10.3389/fneur.2023.1281412)
Supplement: Supplementary file 1 [file Data_Sheet_1.zip › fneur.2023.1281412 - Datasheet attached/Data Sheet 2 (1).pdf]

CARE case report guidelines

CARE Checklist of information to include when writing a case report

(cc) BY-NC-ND

Topic	Item	Checklist item description	Reported on Line
Title	1	The diagnosis or intervention of primary focus followed by the words "case report"	Page 01
Key Words	2	2 to 5 key words that identify diagnoses or interventions in this case report, including "case report"	Page 01
Abstract (no references)	3a	Introduction: What is unique about this case and what does it add to the scientific literature?	Page 01-02
	3b	Main symptoms and/or important clinical findings	Page 02-03
	3c	The main diagnoses, therapeutic interventions, and outcomes	Page 02-03
	3d	Conclusion—What is the main "take-away" lesson(s) from this case?	Page 05
Introduction	4	One or two paragraphs summarizing why this case is unique (**may include references**)	Page 01-03
Patient Information	5a	De-identified patient specific information.	Page 02-03
	5b	Primary concerns and symptoms of the patient.	Page 02-03
	5c	Medical, family, and psycho-social history including relevant genetic information	Page 02-03
	5d	Relevant past interventions with outcomes	Page 02-03
Clinical Findings	6	Describe significant physical examination (PE) and important clinical findings.	Page 02-03
Timeline	7	Historical and current information from this episode of care organized as a timeline	No
Diagnostic Assessment	8a	Diagnostic testing (such as PE, laboratory testing, imaging, surveys).	Page 02-03
	8b	Diagnostic challenges (such as access to testing, financial, or cultural)	No
	8c	Diagnosis (including other diagnoses considered)	Page 02-03
	8d	Prognosis (such as staging in oncology) where applicable	Page 02-03
Therapeutic Intervention	9a	Types of therapeutic intervention (such as pharmacologic, surgical, preventive, self-care)	Page 02-03
	9b	Administration of therapeutic intervention (such as dosage, strength, duration)	Page 02-03
	9c	Changes in therapeutic intervention (with rationale)	No
Follow-up and Outcomes	10a	Clinician and patient-assessed outcomes (if available)	Page 02-03
	10b	Important follow-up diagnostic and other test results	Page 02-03
	10c	Intervention adherence and tolerability (How was this assessed?)	Page 02-03
	10d	Adverse and unanticipated events	No
Discussion	11a	A scientific discussion of the strengths AND limitations associated with this case report	Page 03, 05
	11b	Discussion of the relevant medical literature **with references**.	Page 03, 05
	11c	The scientific rationale for any conclusions (including assessment of possible causes)	Page 05
	11d	The primary "take-away" lessons of this case report (without references) in a one paragraph conclusion	Page 05
Patient Perspective	12	The patient should share their perspective in one to two paragraphs on the treatment(s) they received	No
Informed Consent	13	Did the patient give informed consent? Please provide if requested	**Yes** ☑ **No** ☐
